# Supplementary material for: Intraurban Geographic and Socioeconomic Inequalities of Mortality in Four Cities in Colombia
Source: Int J Environ Res Public Health. 2023 Jan 5;20(2):992. doi: 10.3390/ijerph20020992 (PMC9859133; doi:10.3390/ijerph20020992)

Supplementary material

Intraurban Geographic and Socioeconomic Inequalities of Mortality in Four Cities in Colombia

Laura A. Rodriguez-Villamizar [1,*], Diana Marín [2], Juan Gabriel Piñeros-Jiménez [3], Oscar Alberto Rojas-Sánchez [4], Jesus Serrano-Lomelin [5] and Victor Herrera [1]

[1] Department of Public Health, School of Medicine, Universidad Industrial de Santander, Bucaramanga 681012, Colombia
[2] School of Medicine, Universidad Pontificia Bolivariana, Medellin 050031, Colombia
[3] School of Public Health, Universidad de Antioquia, Medellin 050010, Colombia
[4] Division of Public Health Research, Project Bank Team, National Institute of Health-INS Colombia, Bogotá 110221, Colombia
[5] Department of Public Health, Queen's University, Kingston, ON K7L 3N6, Canada
* Correspondence: laurovi@uis.edu.co

Contents

Supplementary Tables...3

Table S1. Multivariable Poisson regression models on the association between smoothed Bayesian mortality rates and quintiles of multidimensional poverty index (MPI) and Concentration Index at Extremes (CIE) for selected cities in Colombia, 2015-2019 ... 3

Table S2. Characteristics of Moran Eigenvector Spatial filter by cause of death and city ... 7

Table S3. Multivariable Poisson regression models on the association between smoothed Bayesian mortality rates for all deaths and aggregated socioeconomic variables with spatial filter for selected cities in Colombia, 2015-2019 ... 8

Supplementary Figures...9

Figure S1. Location of cities included in the study, Colombia 2015-2019. ... 9

Figure S2. Bayesian Mortality Rates for circulatory deaths by city in Colombia 2015-2019 ... 10

Figure S3. Bayesian Mortality Rates for respiratory deaths by city in Colombia 2015-2019 ... 11

Figure S4. Bayesian Mortality Rates for cancer/blood deaths by city in Colombia 2015-2019 ... 12

Figure S5. Concentration index (CI) curve for circulatory deaths by Multidimensional Poverty Index (MPI) for four cities in Colombia 2015-2019 ... 13

Figure S6. Concentration index (CI) curve for respiratory deaths by Multidimensional Poverty Index (MPI) for four cities in Colombia 2015-2019 ... 14

Figure S7. Concentration index (CI) curve for cancer/blood deaths by Multidimensional Poverty Index (MPI) for four cities in Colombia 2015-2019 ... 15

Figure S8. Geographic gradient of Bayesian mortality rates for circulatory deaths across census sectors by the spatial filter quintiles by cities in Colombia, 2015-2019 ... 16

Figure S9. Geographic gradient of Bayesian mortality rates for respiratory deaths across census sectors by the spatial filter quintiles by cities in Colombia, 2015-2019 ... 17

Figure S10. Geographic gradient of Bayesian mortality rates for cancer/blood deaths across census sectors by the spatial filter quintiles by cities in Colombia, 2015-2019. ... 18

Figure S11. Spatial Filter distribution for circulatory deaths at census sector level by city in Colombia, 2015-2019 ... 19

Figure S12. Spatial Filter distribution for respiratory deaths at census sector level by city in Colombia, 2015-2019 ... 20

Figure S13. Spatial Filter distribution for cancer/blood deaths at census sector level by city in Colombia, 2015-2019 ... 21

Supplementary Tables

Table S1. Multivariable Poisson regression models on the association between smoothed Bayesian mortality rates and quintiles of multidimensional poverty index (MPI) and Concentration Index at Extremes (CIE) for selected cities in Colombia, 2015-2019

	Model 3 using MPI		Model 4 using CIE	
**City-Model estimations**	**IRR**	**95% CI**	**IRR**	**95% CI**
**Barranquilla**				
Circulatory deaths				
Index -Q1	Reference		Reference	
Q2	1.08	0.88-1.31	1.09	0.89-1.34
Q3	1.1	0.92-1.33	1.18	0.99-1.41
Q4	1.07	0.76-1.51	1.04	0.79-1.36
Q5	1.28	0.85-1.91	**1.35**	**1.02-1.79**
>25% adults aged 60 or more years	**1.51**	**1.28-1.80**	**1.49**	**1.28-1.75**
>48% male sex	1.01	0.72-1.43	1.01	0.82-1.25
Spatial Filter	**1.57**	**1.41-1.75**	**1.59**	**1.42-1.78**
Adjusted R-squared	0.32		0.34	
Moran´s Index of residuals (p-value)	.-0.046 (0.788)		.-0.046 (0.889)	
Respiratory deaths				
Index -Q1	Reference		Reference	
Q2	1.08	0.87-1.33	1.11	0.91-1.35
Q3	1.11	0.90-1.36	**1.26**	**1.08-1.47**
Q4	0.89	0.67-1.21	0.94	0.73-1.20
Q5	1.12	0.78-1.62	1.28	0.98-1.65
>25% adults aged 60 or more years	**1.53**	**1.29-1.81**	**1.52**	**1.29-1.81**
>48% male sex	1.08	0.78-1.49	1.01	0.81-1.27
Spatial Filter	**1.62**	**1.44-1.82**	**1.65**	**1.47-1.85**
Adjusted R-squared	0.28		0.3	
Moran´s Index of residuals (p-value)	.-0.037 (0.817)		.-0.025 (0.716)	
Cancer deaths				
Index -Q1	Reference		Reference	
Q2	1.04	0.85-1.27	1.06	0.88-1.28
Q3	1.02	0.85-1.21	1.07	0.88-1.28
Q4	0.82	0.62-1.07	0.87	0.69-1.09
Q5	0.94	0.67-1.31	1.03	0.79-1.35
>25% adults aged 60 or more years	**1.55**	**1.33-1.82**	**1.57**	**1.34-1.82**
>48% male sex	1.12	0.83-1.50	1.05	0.87-1.26
Spatial Filter	**1.42**	**1.29-1.55**	**1.42**	**1.29-1.55**
Adjusted R-squared	0.33		0.33	
Moran´s Index of residuals (p-value)	.-0.011 (0.576)		.-0.003 (0.483)	

Table S1 (Continuation). Multivariable Poisson regression models on the association between smoothed Bayesian mortality rates and quintiles of multidimensional poverty index (MPI) and Concentration Index at Extremes (CIE) for selected cities in Colombia, 2015-2019

	**Model 3 using MPI**		**Model 4 using CIE**	
**City-Model estimations**	**IRR**	**95% CI**	**IRR**	**95% CI**
**Bogotá**				
Circulatory deaths				
Index -Q1	Reference		Reference	
Q2	**1.23**	**1.05-1.44**	0.97	0.82-1.15
Q3	**1.18**	**1.00-1.39**	0.94	0.82-1.09
Q4	1.02	0.85-1.22	0.88	0.75-1.04
Q5	0.97	0.81-1.18	**0.82**	**0.69-0.98**
>25% adults aged 60 or more years	**1.5**	**1.28-1.75**	**1.35**	**1.17-1.56**
>48% male sex	**1.2**	**1.07-1.35**	**1.19**	**1.06-1.33**
Spatial Filter	**1.67**	**1.46-1.90**	1.65	1.43-1.89
Pseudo R-squared	0.31		0.3	
Moran´s Index of residuals (p-value)	.-0.06 (0.996)		.-0.061 (0.993)	
Respiratory deaths				
Index -Q1	Reference		Reference	
Q2	**1.21**	**1.05-1.39**	0.97	0.84-1.13
Q3	**1.17**	**1.01-1.37**	0.98	0.85-1.11
Q4	1.12	0.95-1.32	0.97	0.83-1.12
Q5	1.04	0.88-1.24	0.91	0.77-1.06
>25% adults aged 60 or more years	**1.39**	**1.21-1.59**	**1.27**	**1.11-1.44**
>48% male sex	**1.2**	**1.07-1.34**	**1.19**	**1.08-1.32**
Spatial Filter	**1.99**	**1.77-2.24**	**1.98**	**1.76-2.25**
Pseudo R-squared	0.27		0.26	
Moran´s Index of residuals (p-value)	.-0.065 (0.998)		.-0.064 (0.995)	
Cancer deaths				
Index -Q1	Reference		Reference	
Q2	**1.14**	**1.02-1.26**	0.99	0.89-1.11
Q3	1.06	0.94-1.21	0.95	0.85-1.07
Q4	0.96	0.84-1.09	0.91	0.81-1.03
Q5	0.88	0.77-1.01	**0.85**	**0.74-0.97**
>25% adults aged 60 or more years	**1.41**	**1.27-1.56**	**1.34**	**1.22-1.47**
>48% male sex	**1.12**	**1.03-1.22**	**1.09**	**1.00-1.18**
Spatial Filter	**1.65**	**1.49-1.83**	**1.65**	**1.47-1.84**
Pseudo R-squared	0.28		0.27	
Moran´s Index of residuals (p-value)	0.040 (0.039)		0.025 (0.124)	

Table S1 (Continuation). Multivariable Poisson regression models on the association between smoothed Bayesian mortality rates and quintiles of multidimensional poverty index (MPI) and Concentration Index at Extremes (CIE) for selected cities in Colombia, 2015-2019

	Model 3 using MPI		Model 4 using CIE	
**City-Model estimations**	**IRR**	**95% CI**	**IRR**	**95% CI**
**Cali**				
Circulatory deaths				
Index -Q1	Reference		Reference	
Q2	0.94	0.78-1.13	0.94	0.77-1.14
Q3	1.13	0.95-1.35	1.11	0.91-1.34
Q4	**1.35**	**1.09-1.67**	**1.32**	**1.03-1.69**
Q5	**1.35**	**1.11-1.64**	**1.27**	**1.02-1.59**
>25% adults aged 60 or more years	**1.32**	**1.14-1.55**	**1.29**	**1.11-1.52**
>48% male sex	**0.69**	**0.56-0.85**	**0.73**	**0.59-0.89**
Spatial Filter	**1.89**	**1.68-2.14**	**1.89**	**1.67-2.15**
Pseudo R-squared	0.31		0.3	
Moran´s Index of residuals (p-value)	.-0.047 (0.931)		.-0.053 (0.949)	
Respiratory deaths				
Index -Q1	Reference		Reference	
Q2	0.91	0.76-1.04	0.95	0.79-1.15
Q3	1.12	0.96-1.31	1.1	0.91-1.33
Q4	1.13	0.96-1.33	1.12	0.91-1.38
Q5	**1.21**	**1.00-1.45**	1.17	0.93-1.47
>25% adults aged 60 or more years	**1.44**	**1.26-1.65**	**1.41**	**1.22-1.63**
>48% male sex	**0.81**	**0.67-0.98**	0.83	0.69-1.00
Spatial Filter	**1.7**	**1.53-1.88**	**1.69**	**1.52-1.87**
Pseudo R-squared	0.27		0.27	
Moran´s Index of residuals (p-value)	.-0.054 (0.972)		.-0.056 (0.965)	
Cancer deaths				
Index -Q1	Reference		Reference	
Q2	0.92	0.79-1.07	0.94	0.79-1.11
Q3	0.98	0.83-1.14	1.01	0.84-1.19
Q4	1.09	0.93-1.28	1.04	0.86-1.26
Q5	1.01	0.83-1.21	0.97	0.78-1.20
>25% adults aged 60 or more years	**1.3**	**1.15-1.48**	**1.27**	**1.12-1.45**
>48% male sex	0.79	0.62-1.02	0.83	0.65-1.05
Spatial Filter	**1.73**	**1.49-1.99**	**1.72**	**1.49-1.99**
Pseudo R-squared	0.22		0.22	
Moran´s Index of residuals (p-value)	.-0.60 (0.971)		.-0.067 (0.983)	

Table S1 (Continuation). Multivariable Poisson regression models on the association between smoothed Bayesian mortality rates and quintiles of multidimensional poverty index (MPI) and Concentration Index at Extremes (CIE) for selected cities in Colombia, 2015-2019

	Model 3 using MPI		Model 4 using CIE	
**City-Model estimations**	**IRR**	**95% CI**	**IRR**	**95% CI**
**Medellín**				
Circulatory deaths				
Index -Q1	Reference		Reference	
Q2	**1.21**	**1.03-1.41**	0.97	0.81-1.17
Q3	**1.35**	**1.11-1.64**	1.17	0.95-1.43
Q4	1.23	0.99-1.53	1.09	0.88-1.35
Q5	0.93	0.72-1.20	0.82	0.63-1.07
>25% adults aged 60 or more years	**1.76**	**1.49-2.08**	**1.62**	**1.39-1.89**
>48% male sex	1.18	0.99-1.41	1.16	0.98-1.38
Spatial Filter	**1.21**	**1.12-1.29**	**1.23**	**1.15-1.32**
Pseudo R-squared	0.33		0.32	
Moran´s Index of residuals (p-value)	.-0.046 (0.878)		.-0.027 (0.753)	
Respiratory deaths				
Index -Q1	Reference		Reference	
Q2	1.11	0.93-1.32	0.97	0.79-1.19
Q3	1.19	0.95-1.49	1.08	0.88-1.33
Q4	1.01	0.79-1.28	0.94	0.74-1.18
Q5	0.78	0.59-1.02	**0.72**	**0.55-0.95**
>25% adults aged 60 or more years	**1.79**	**1.48-2.16**	**1.71**	**1.47-1.98**
>48% male sex	1.09	0.95-1.28	1.08	0.93-1.27
Spatial Filter	**1.25**	**1.16-1.35**	**1.25**	**1.15-1.37**
Pseudo R-squared	0.32		0.31	
Moran´s Index of residuals (p-value)	.-0.037 (0.802)		.-0.025 (0.685)	
Cancer deaths				
Index -Q1	Reference		Reference	
Q2	1.09	0.94-1.26	1.06	0.86-1.32
Q3	1.25	1.02-1.55	1.09	0.90-1.31
Q4	1.12	0.84-1.49	0.98	0.79-1.21
Q5	0.79	0.60-1.03	**0.71**	**0.55-0.91**
>25% adults aged 60 or more years	**1.81**	**1.44-2.82**	**1.63**	**1.42-1.88**
>48% male sex	**1.28**	**1.06-1.53**	**1.29**	**1.06-1.58**
Spatial Filter	**1.07**	**1.03-1.11**	**1.07**	**1.03-1.11**
Pseudo R-squared	0.33		0.32	
Moran´s Index of residuals (p-value)	0.032 (0.119)		0.024 (0.188)	

Table S2. Characteristics of Moran Eigenvector Spatial filter by cause of death and city

All deaths

City	No. Eigenvectors extracted	R2	Moran´s Index	p-value
Barranquilla	12 of 29	0.34487	0.82219	<0,001
Bogotá	25 of 86	0.13446	0.86901	<0,001
Cali	20 of 74	0.30668	0.84207	<0,001
Medellín	17 of 51	0.35062	0.82366	<0,001

Circulatory

City	No. Eigenvectors extracted	R2	Moran´s Index	p-value
Barranquilla	10 of 27	0.32294	0.79063	<0,001
Bogotá	55 of 143	0.32466	0.78623	<0,001
Cali	19 of 74	0.2928	0.8499	<0,001
Medellín	12 of 45	0.26446	0.83786	<0,001

Respiratory

City	No. Eigenvectors extracted	R2	Moran´s Index	p-value
Barranquilla	11 of 29	0.33312	0.84477	<0,001
Bogotá	57 of 142	0.32011	0.80284	<0,001
Cali	20 of 73	0.30552	0.82183	<0,001
Medellín	11 of 44	0.22875	0.87263	<0,001

Cancer/blood

City	No. Eigenvectors extracted	R2	Moran´s Index	p-value
Barranquilla	12 of 30	0.32455	0.82498	<0,001
Bogotá	46 of 141	0.32006	0.83975	<0,001
Cali	15 of 64	0.23908	0.84819	<0,001
Medellín	18 of 52	0.33176	0.82368	<0,001

Table S3. Multivariable Poisson regression models on the association between smoothed Bayesian mortality rates for all deaths and aggregated socioeconomic variables with spatial filter for selected cities in Colombia, 2015-2019

	**Barranquilla**		**Bogotá**		**Cali**		**Medellín**	
**City-Model estimations**	**IRR**	**95% CI**	**IRR**	**95% CI**	**IRR**	**95% CI**	**IRR**	**95% CI**
>25% adults aged 60 or more years	**1.77**	**1.47-2.12**	**1.78**	**1.55-2.04**	**1.29**	**1.13-1.47**	**1.69**	**1.47-1.95**
>48% male sex	1.07	0.86-1.32	1.06	0.95-1.19	**0.81**	**0.65-0.98**	**1.21**	**1.01-1.44**
>15% adults with primary level or no education	0.95	0.75-1.21	**1.16**	**1.03-1.32**	**1.37**	**1.19-1.59**	1.06	0.85-1.32
>6% adults unemployed looking for work	1.05	0.88-1.23	**0.84**	**0.75-0.93**	**0.86**	**0.75-0.98**	0.88	0.76-1.019
Low household strata 1-3	1.16	0.97-1.37	**1.19**	**1.03-1.36**	0.97	0.82-1.16	1.04	0.85-1.28
Spatial Filter	**1.36**	**1.25-1.47**	**1.48**	**1.36-1.61**	**1.67**	**1.51-1.86**	**1.06**	**1.03-1.10**
Pseudo R-squared	0.4		0.29		0.39		0.3	
Moran´s Index of residuals (p-value)	0.044 (0.136)		.-0.031 (0.927)		.-0.078 (0.997)		.-0.009 (0.528)	

Supplementary Figures

Figure S1. Location of cities included in the study, Colombia 2015-2019.

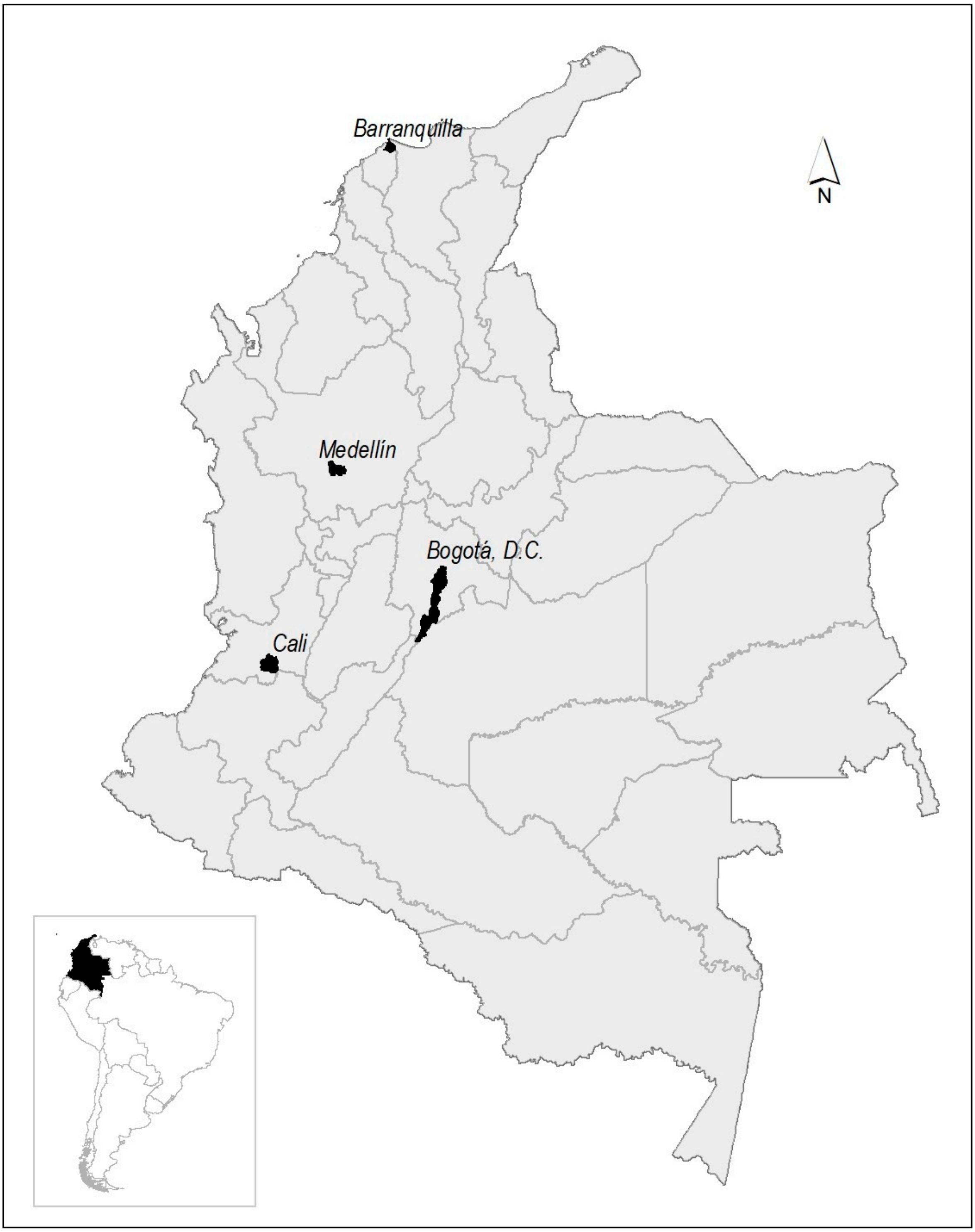

Note: Gray outlines define department borders.

Figure S2. Bayesian Mortality Rates for circulatory deaths by city in Colombia 2015-2019

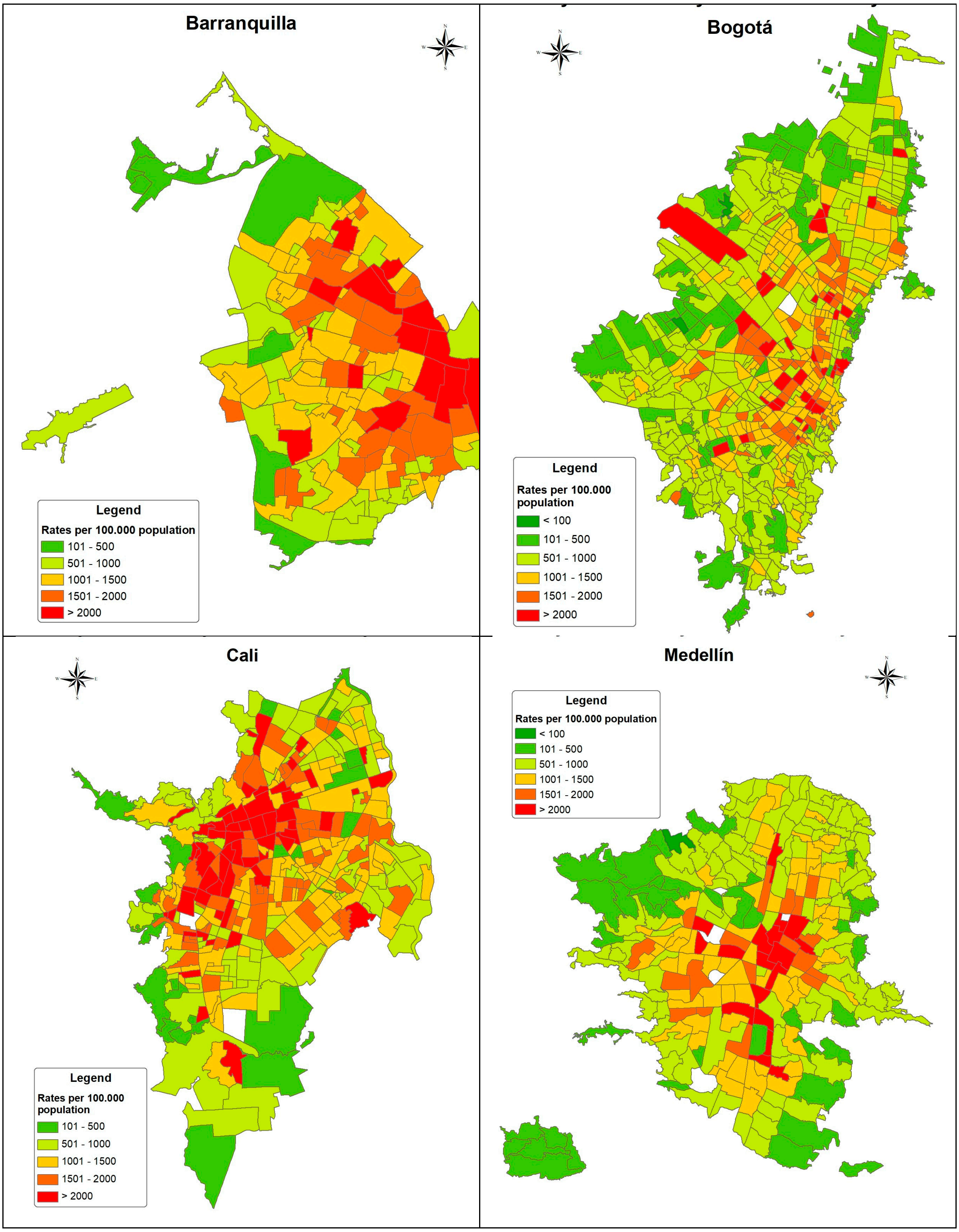

Figure S3. Bayesian Mortality Rates for respiratory deaths by city in Colombia 2015-2019

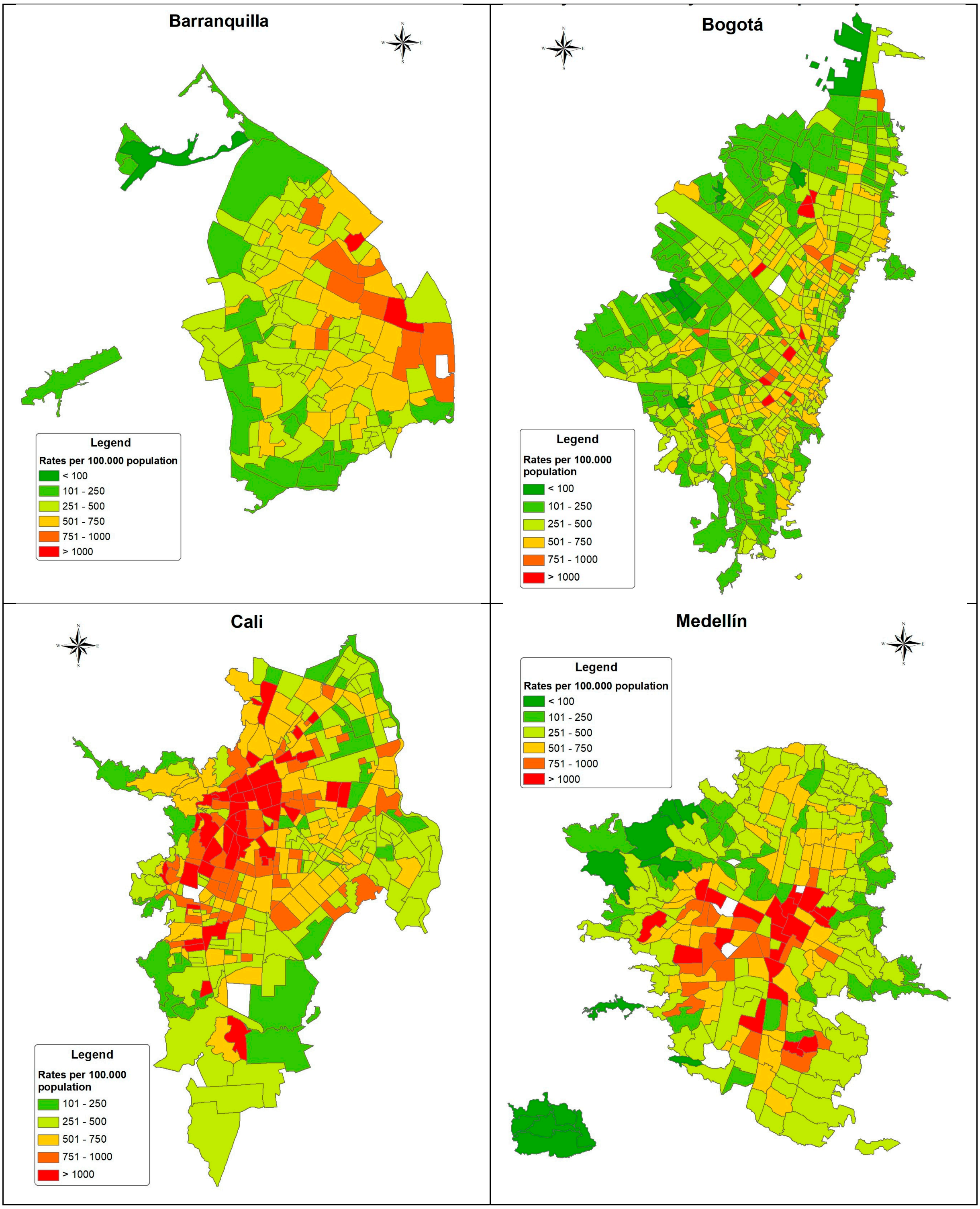

Figure S4. Bayesian Mortality Rates for cancer/blood deaths by city in Colombia 2015-2019

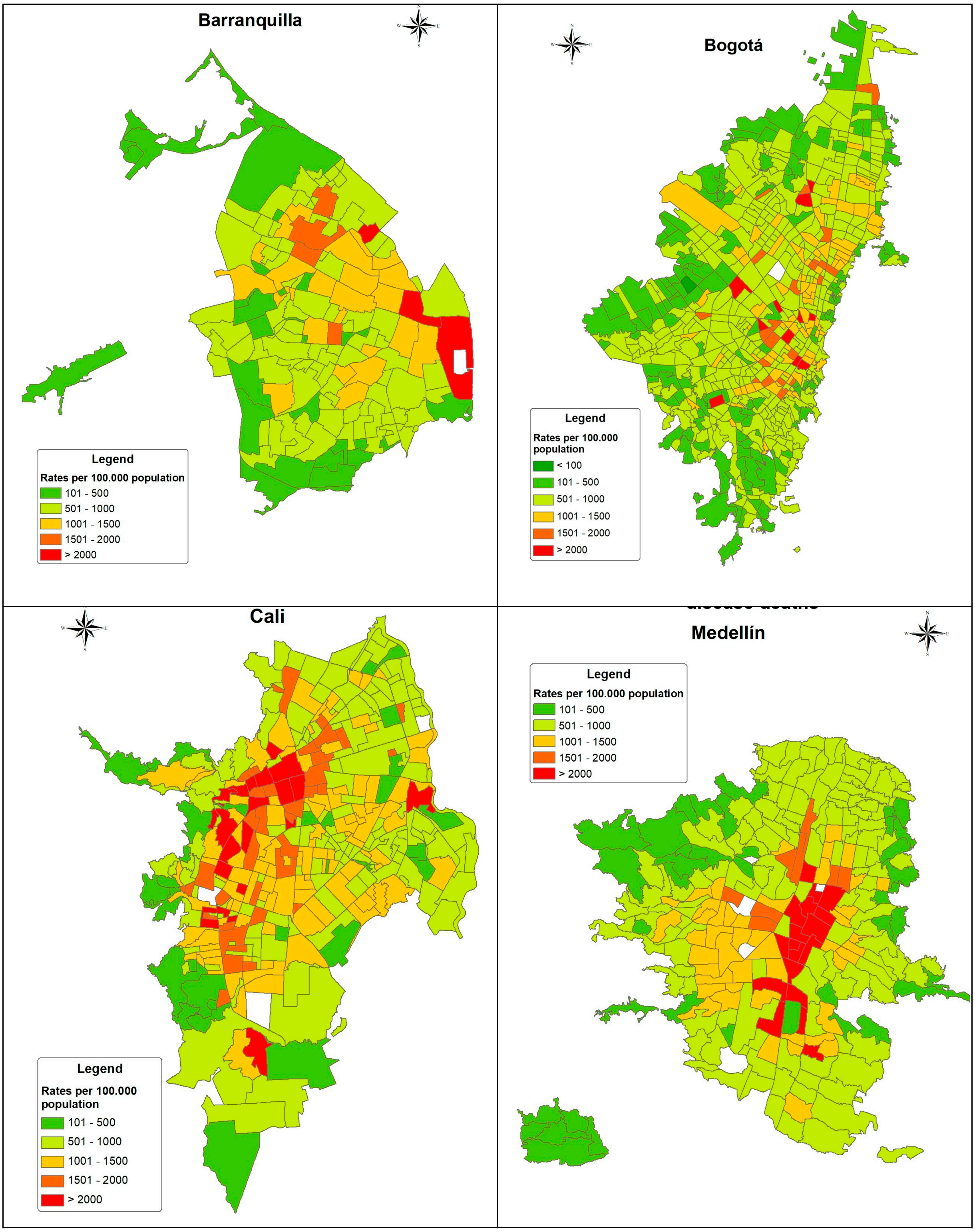

Figure S5. Concentration index (CI) curve for circulatory deaths by Multidimensional Poverty Index (MPI) for four cities in Colombia 2015-2019

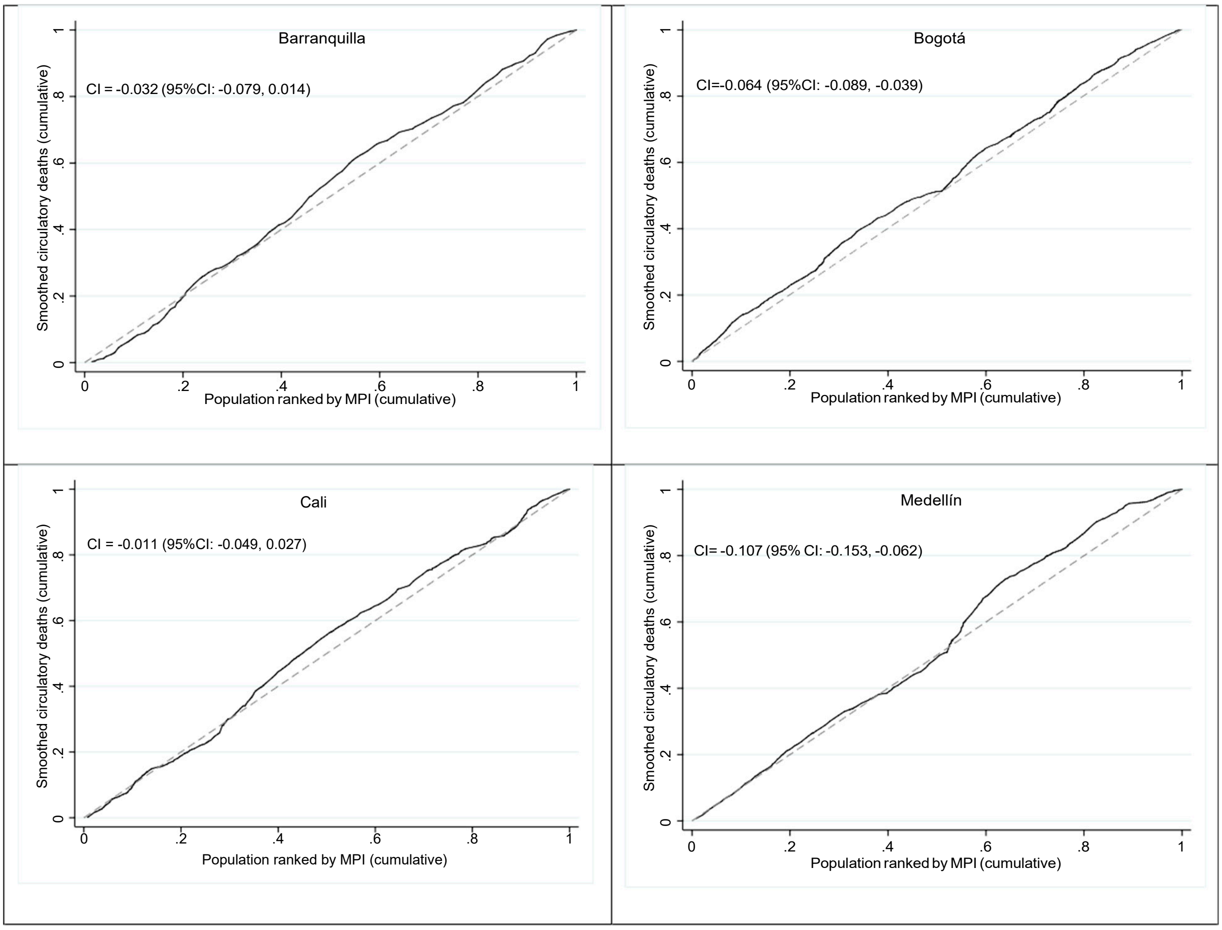

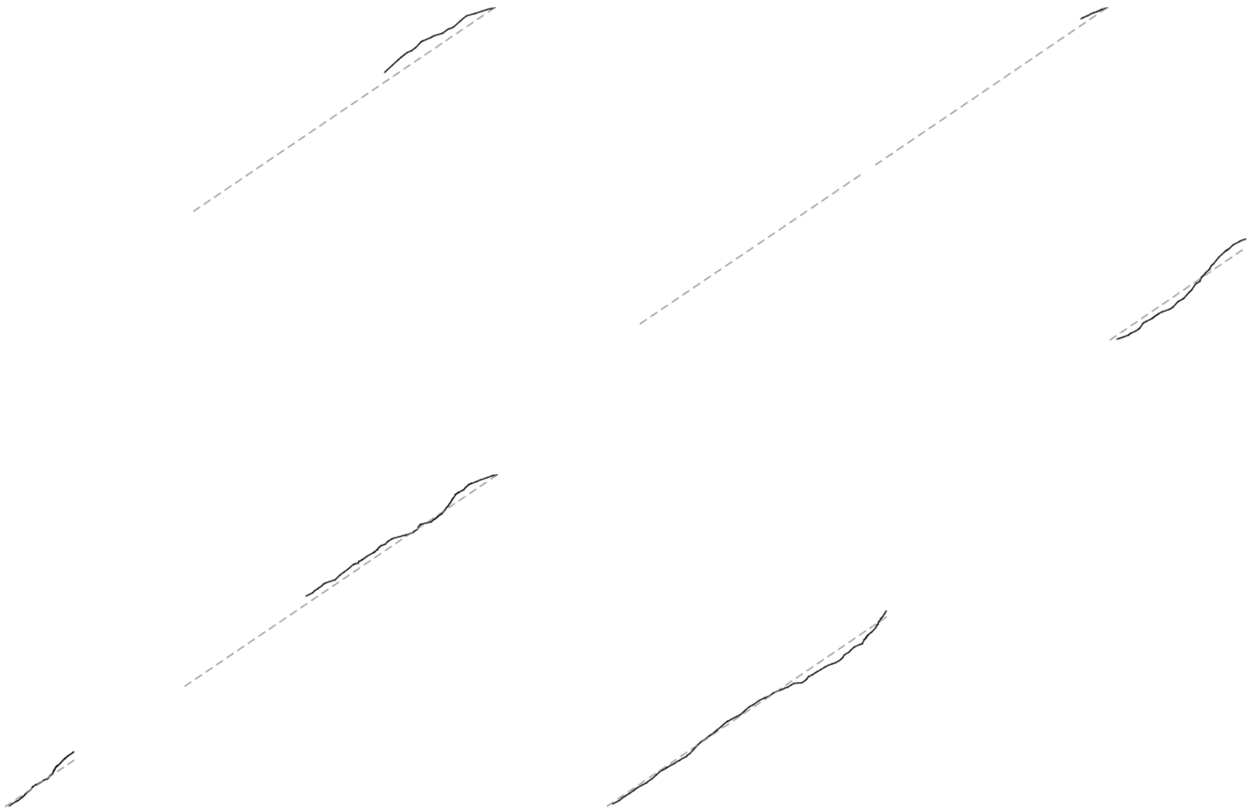

Figure S7. Concentration index (CI) curve for cancer/blood deaths by Multidimensional Poverty Index (MPI) for four cities in Colombia 2015-2019

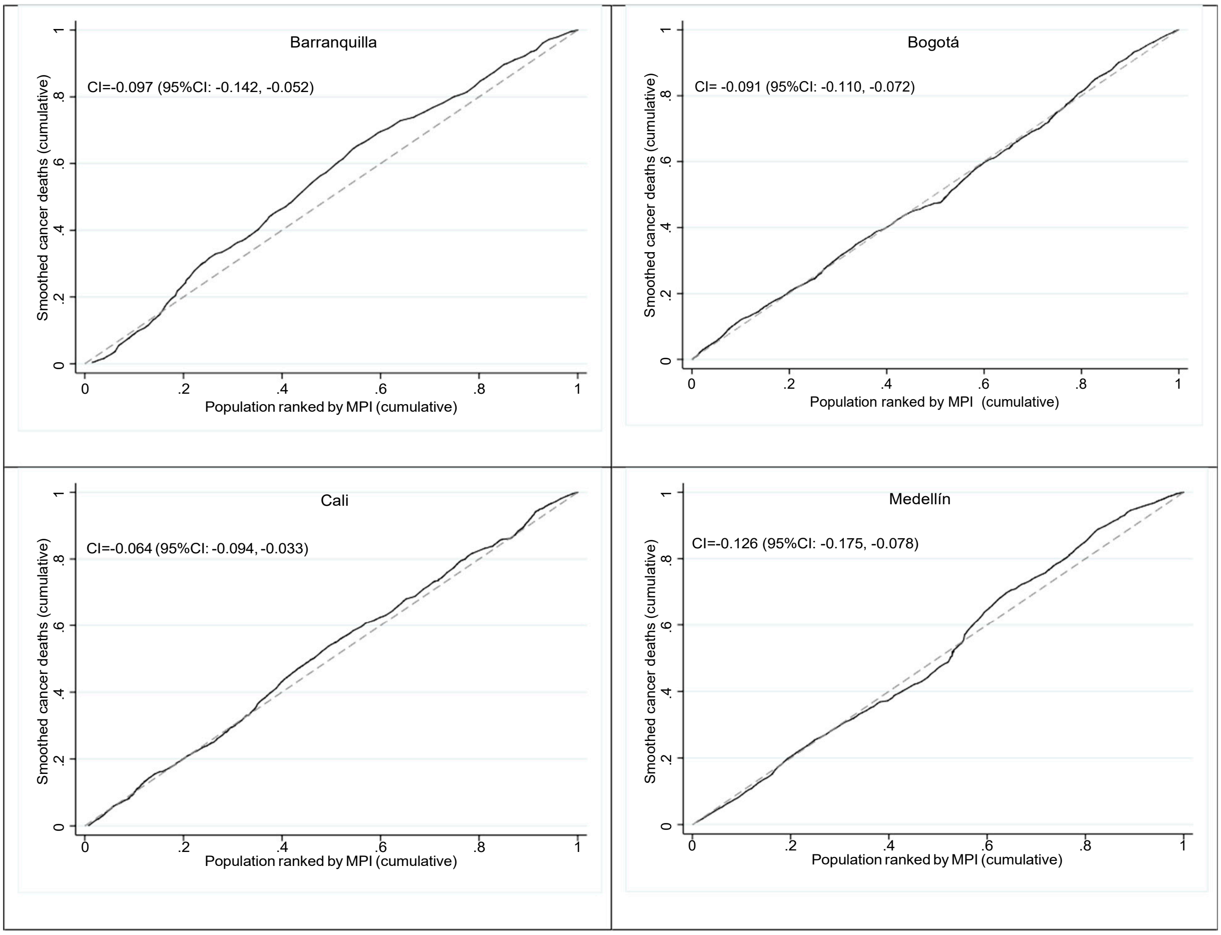

Figure S8. Geographic gradient of Bayesian mortality rates for circulatory deaths across census sectors by the spatial filter quintiles by cities in Colombia, 2015-2019.

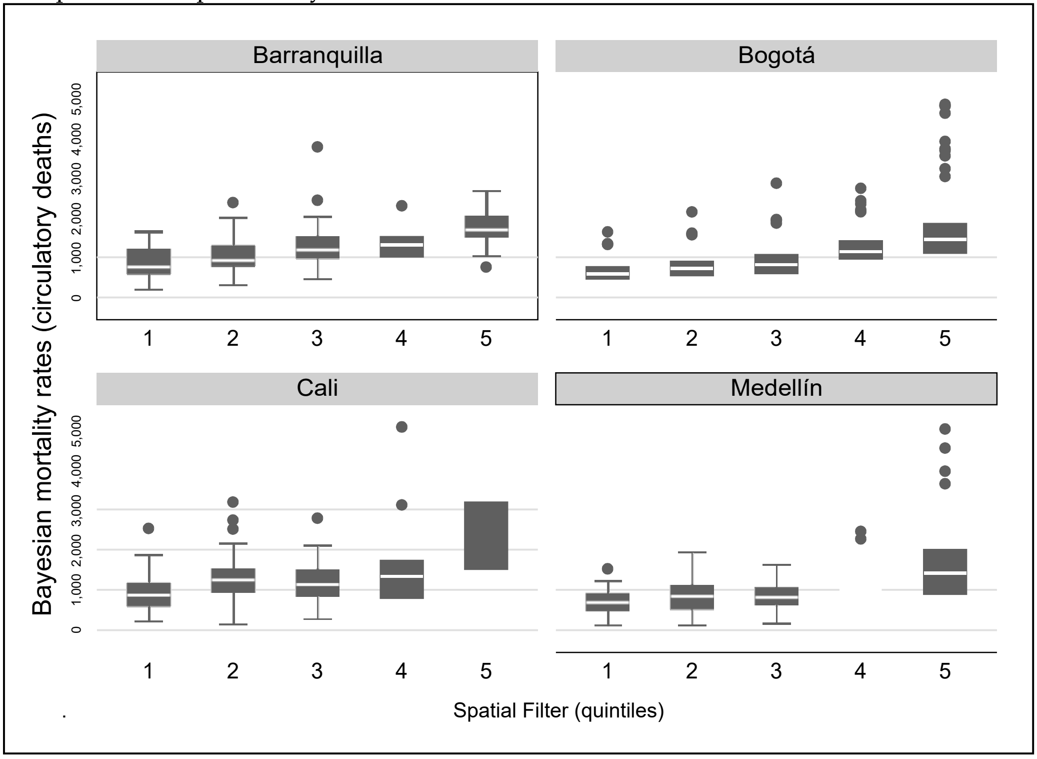

Note: Quintiles were split according to the spatial filter range values for each city.

Figure S9. Geographic gradient of Bayesian mortality rates for respiratory deaths across census sectors by the spatial filter quintiles by cities in Colombia, 2015-2019.

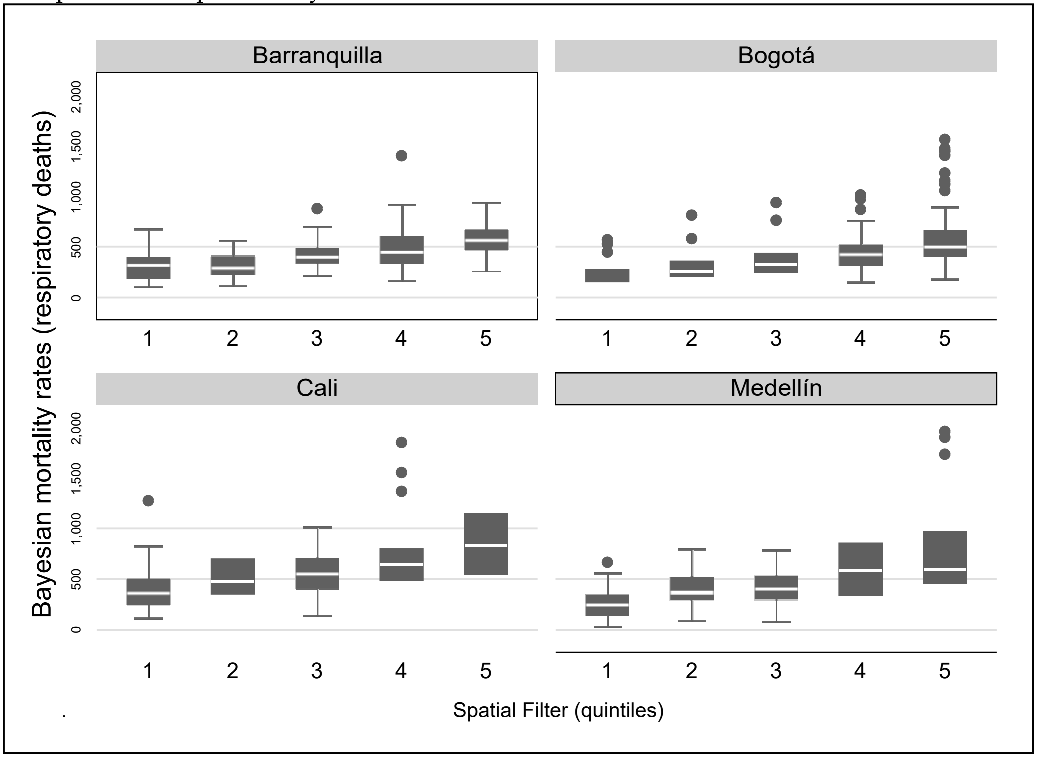

Note: Quintiles were split according to the spatial filter range values for each city.

Figure S10. Geographic gradient of Bayesian mortality rates for cancer/blood deaths across census sectors by the spatial filter quintiles by cities in Colombia, 2015-2019.

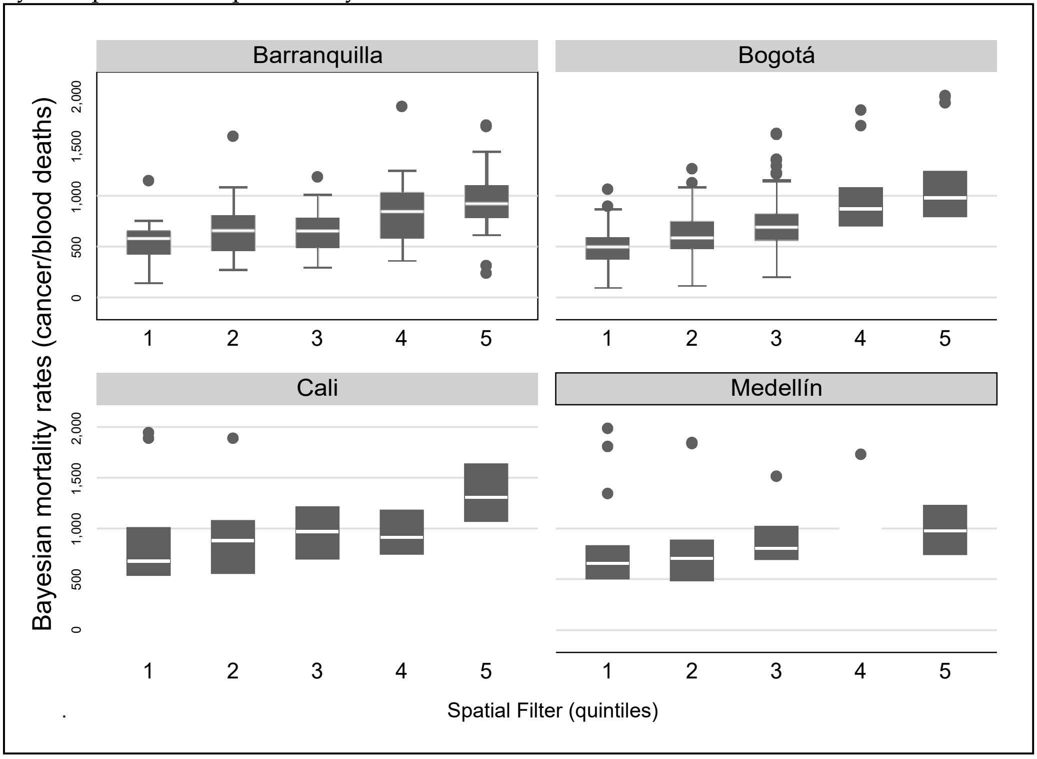

Note: Quintiles were split according to the spatial filter range values for each city.

Figure S11. Spatial Filter distribution for circulatory deaths at census sector level by city in Colombia, 2015-2019

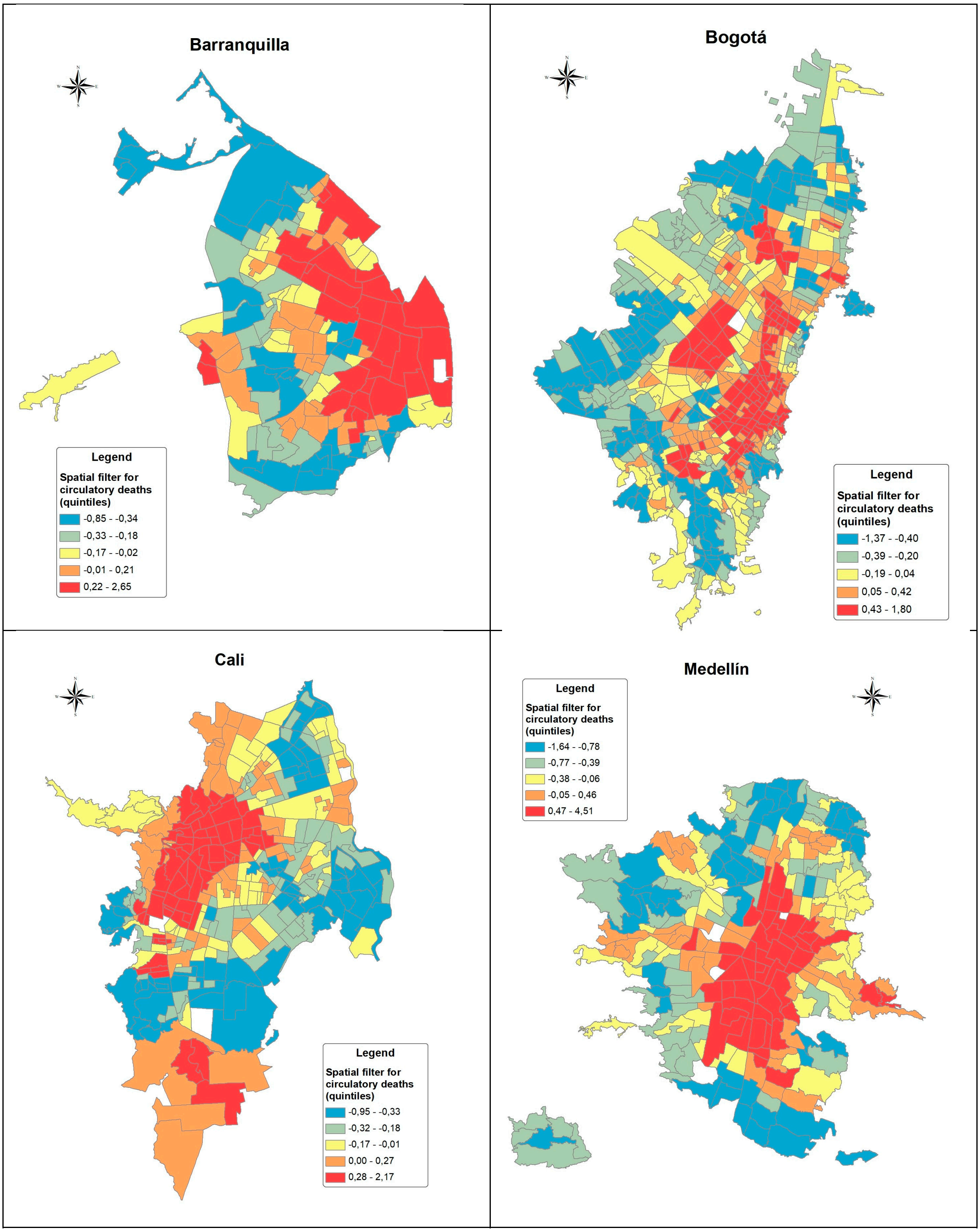

Figure S12. Spatial Filter distribution for respiratory deaths at census sector level by city in Colombia, 2015-2019

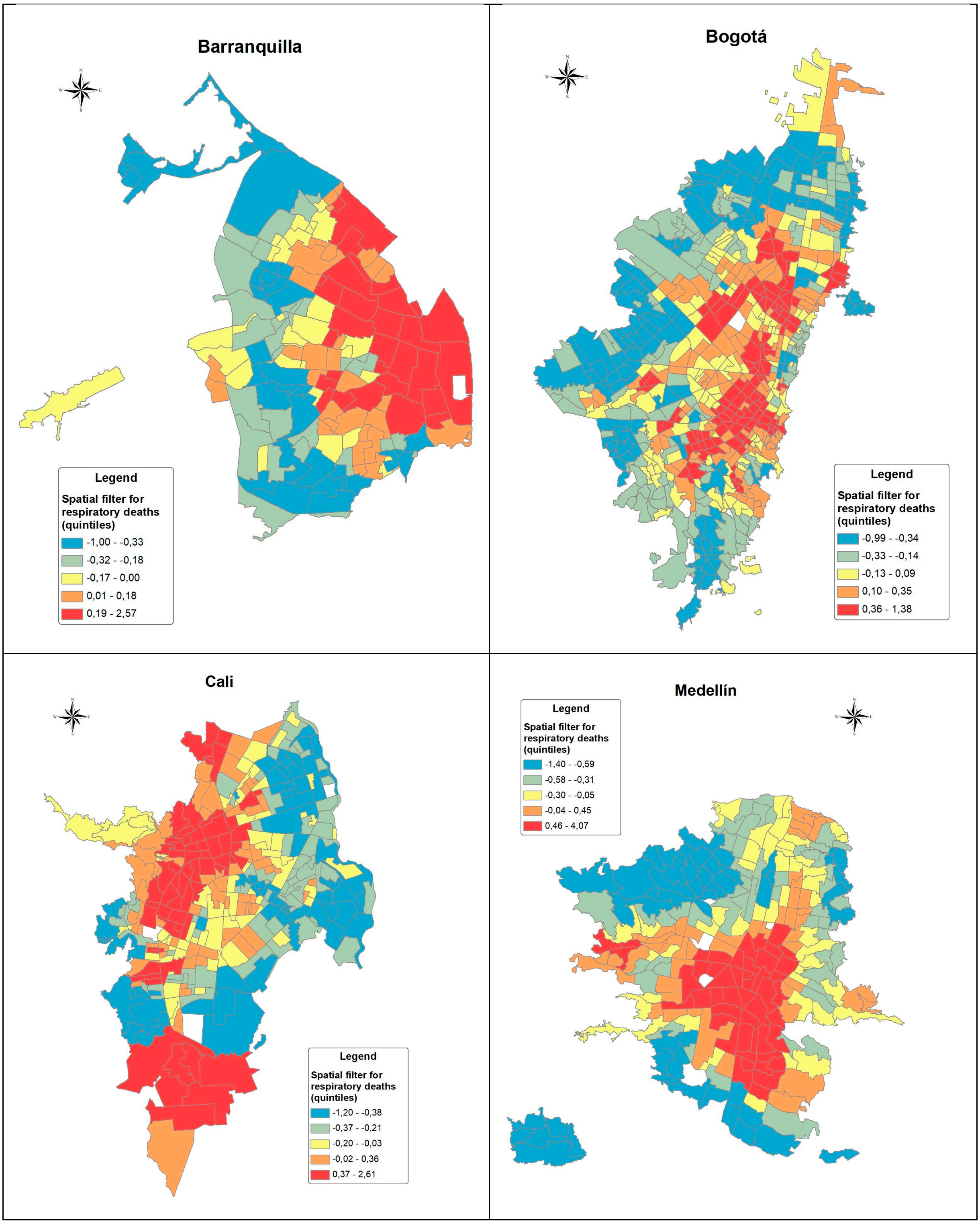

Figure S13. Spatial Filter distribution for cancer/blood deaths at census sector level by city in Colombia, 2015-2019

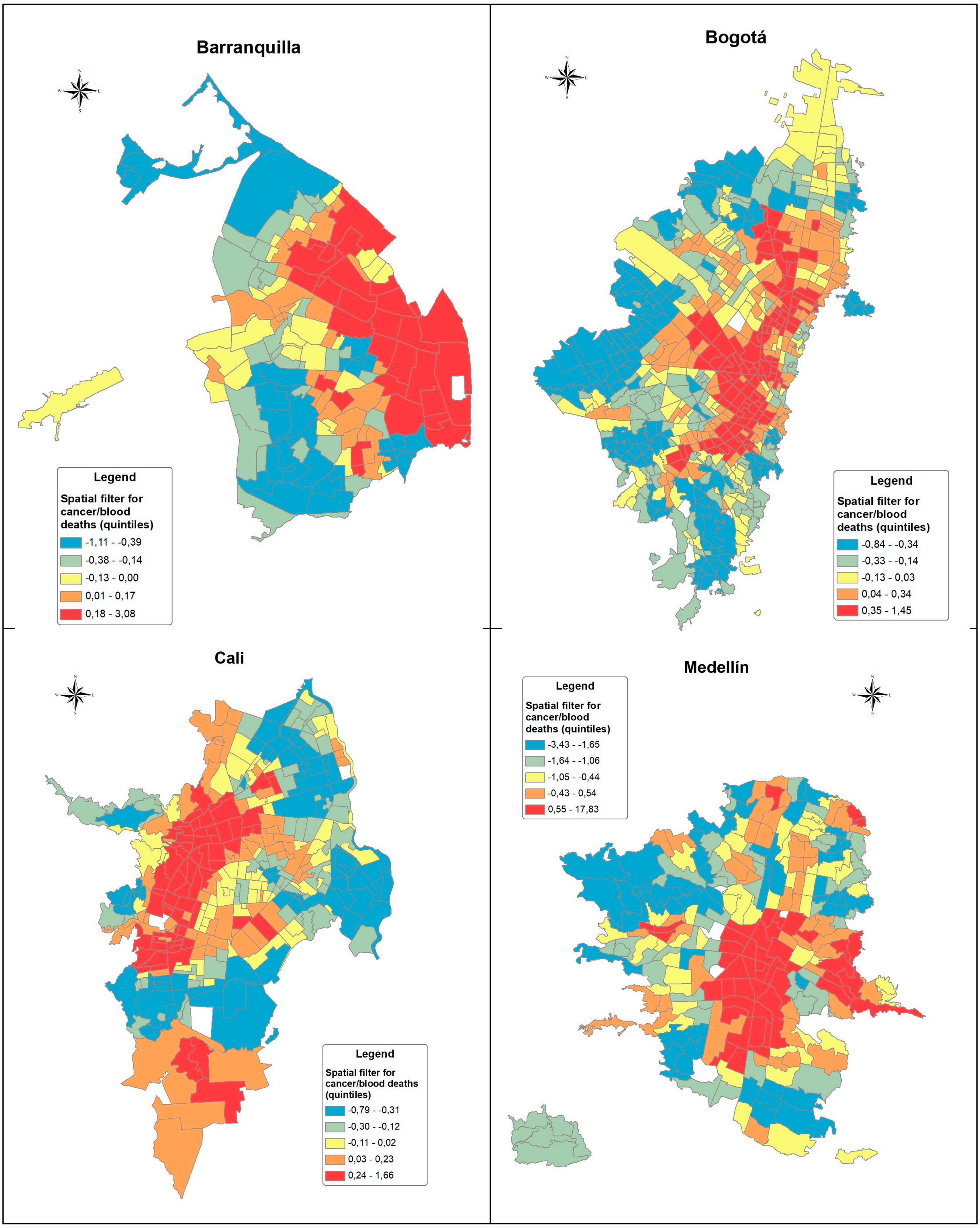

Supplement: Supplementary file 1 [file ijerph-20-00992-s001.zip › ijerph-2080668-supplementary.pdf]
